# Near-zero methane emission from an abandoned boreal peatland pasture based on eddy covariance measurements

**DOI:** 10.1371/journal.pone.0189692

**Published:** 2017-12-18

**Authors:** Mei Wang, Jianghua Wu, Junwei Luan, Peter Lafleur, Huai Chen, Xinbiao Zhu

**Affiliations:** 1 Sustainable Resource Management, Memorial University of Newfoundland, Corner Brook, Canada; 2 School of Geographical Sciences, South China Normal University, Guangzhou, China; 3 International Center for Bamboo and Rattan, Beijing, China; 4 School of the Environment, Trent University, Peterborough, ON, Canada; 5 Key Laboratory of Mountain Ecological Restoration and Bio-resource Utilization & Ecological Restoration Biodiversity Conservation Key Laboratory of Sichuan Province, Chengdu Institute of Biology, Chinese Academy of Sciences, Chengdu, China; 6 Atlantic Forestry Centre, Canadian Forest Service, Natural Resources Canada, Corner Brook, NL, Canada; Yonsei University, REPUBLIC OF KOREA

## Abstract

Although estimates of the annual methane (CH_4_) flux from agriculturally managed peatlands exist, knowledge of controls over the variation of CH_4_ at different time-scales is limited due to the lack of high temporal-resolution data. Here we present CH_4_ fluxes measured from May 2014 to April 2016 using the eddy covariance technique at an abandoned peatland pasture in western Newfoundland, Canada. The goals of the study were to identify the controls on the seasonal variations in CH_4_ flux and to quantify the annual CH_4_ flux. The seasonal variation in daily CH_4_ flux was not strong in the two study years, however a few periods of pronounced emissions occurred in the late growing season. The daily average CH_4_ flux was small relative to other studies, ranging from -4.1 to 9.9 nmol m^-2^ s^-1^ in 2014–15 and from -7.1 to 12.1 nmol m^-2^ s^-1^ in 2015–16. Stepwise multiple regression was used to investigate controls on CH_4_ flux and this analysis found shifting controls on CH_4_ flux at different periods of the growing season. During the early growing season CH_4_ flux was closely related to carbon dioxide fixation rates, suggesting substrate availability was the main control. The peak growing season CH_4_ flux was principally controlled by the CH_4_ oxidation in 2014, where the CH_4_ flux decreased and increased with soil temperature at 50 cm and soil water content at 10 cm, but a contrasting temperature-CH_4_ relation was found in 2015. The late growing season CH_4_ flux was found to be regulated by the variation in water table level and air temperature in 2014. The annual CH_4_ emission was near zero in both study years (0.36 ± 0.30 g CH_4_ m^-2^ yr^-1^ in 2014–15 and 0.13 ± 0.38 g CH_4_ m^-2^ yr^-1^ in 2015–16), but fell within the range of CH_4_ emissions reported for agriculturally managed peatlands elsewhere.

## Introduction

Agricultural drainage is one of the most common management practices in northern peatlands. About 20% of pristine peatlands have been drained for agriculture, forestry, and peat extraction, among which agriculture is now the most widespread human use for peatlands globally [1–5]. Although natural peatlands tend to be carbon dioxide (CO_2_) sinks and methane (CH_4_) sources, they have acted to cool global climate for the past several millennia, sequestrating ~20–30 g C m^-2^ yr^-1^ from the atmosphere, mainly due to slow decomposition rates of peat organic matter under waterlogged conditions [6,7]. Agricultural drainage leads to significant alterations of the hydrology and vegetation of peatlands [8], which can potentially affect their C cycle and their corresponding impact on climate [9]. However, the importance of managed peatlands for global CH_4_ cycling and climate regulation remains uncertain mainly due to the lack of knowledge of CH_4_ flux processes and the underlying mechanisms, which requires reliable high-frequency CH_4_ flux data to resolve [10].

CH_4_ has a significant climate warming potential, about 25 times that of CO_2_ on a 100-year time horizon, and variations in the CH_4_ flux can exert a significant impact on regional and global climate [11]. In peatlands, CH_4_ is produced by methanogenic archaea in the anaerobic layer and is emitted into the atmosphere through diffusion, ebullition and via plant aerenchyma [12]. Ebullition and plant transport are relatively direct paths to the atmosphere, whereas CH_4_ that diffuses through the overlying aerobic soil layer can be partly oxidized to CO_2_ by methanotrophs, reducing the flux considerably [12]. Hence, the dynamics of the CH_4_ flux are determined by the joint effects of the complex and changing processes of CH_4_ production, consumption, and transport, which can vary with many factors, such as water table level, soil water content, temperature, nutrient availability, vegetation composition, pH, redox potential, and physicochemical properties of soils [12–16]. As a result, CH_4_ fluxes usually show great temporal and spatial variability [10,17–19]. A recent review suggested that water table level and temperature are the dominant controls on CH_4_ flux for pristine bogs and fens, but their effects can be partly offset or even overridden by other processes such as vascular plant transport in some wetland types [20].

Drainage for agriculture can inhibit the release of CH_4_ from peatlands by decreasing the thickness of the potential CH_4_ production zone and increasing the thickness of the potential CH_4_ oxidization zone. In contrast, drainage is often associated with cultivation of aerenchymous plants enabling direct transport of CH_4_ from the soil to the atmosphere [19], thus promoting CH_4_ emissions. However, in general, agricultural drainage has been suggested to decrease CH_4_ emission [21]. Yet, knowledge of the dynamic pattern and magnitude of CH_4_ flux for managed peatland systems is limited, especially on short time scales such as hours to days due to a lack of high-frequency measurements. Most earlier studies on CH_4_ flux in agriculturally managed peatlands have been based on weekly or biweekly chamber measurements in European countries such as Finland [4], Sweden [22] and Norway [23,24]. In addition, while studies of controls on CH_4_ flux dynamics for managed peatlands have almost exclusively considered active agricultural management, the effects of long-term abandonment after agricultural conversion is largely unexplored.

In Canada, peatlands cover an area of approximately 1.136 million km^2^, second only to those in Russia [25]. During the past century, extensive areas of Canadian peatlands have been drained for various purposes, such as agriculture, forestry, horticulture and other uses [26]. Agricultural management of peatlands is the most common type of non-harvesting use in Canada [26], with an area of 170,000 km^2^ having been converted for such use, accounting for 15% of the total national resource of peatlands and mires [5]. Although Canada has one of the largest areas of agriculturally managed peatlands, little is known about the magnitude and pattern of CH_4_ exchange in these peatlands. Here, we examine a data set of half-hourly eddy covariance (EC) CH_4_ flux measurements during the period from April, 2014 to June, 2016 at an abandoned peatland-converted pasture in western Newfoundland, Canada. The objectives of the study were: 1) to assess the diel and seasonal variations in CH_4_ flux, 2) to identify the controls on the temporal patterns of the CH_4_ flux and 3) to quantify the annual CH_4_ flux at this site.

## Methods

### Site description

**Fig 1:**
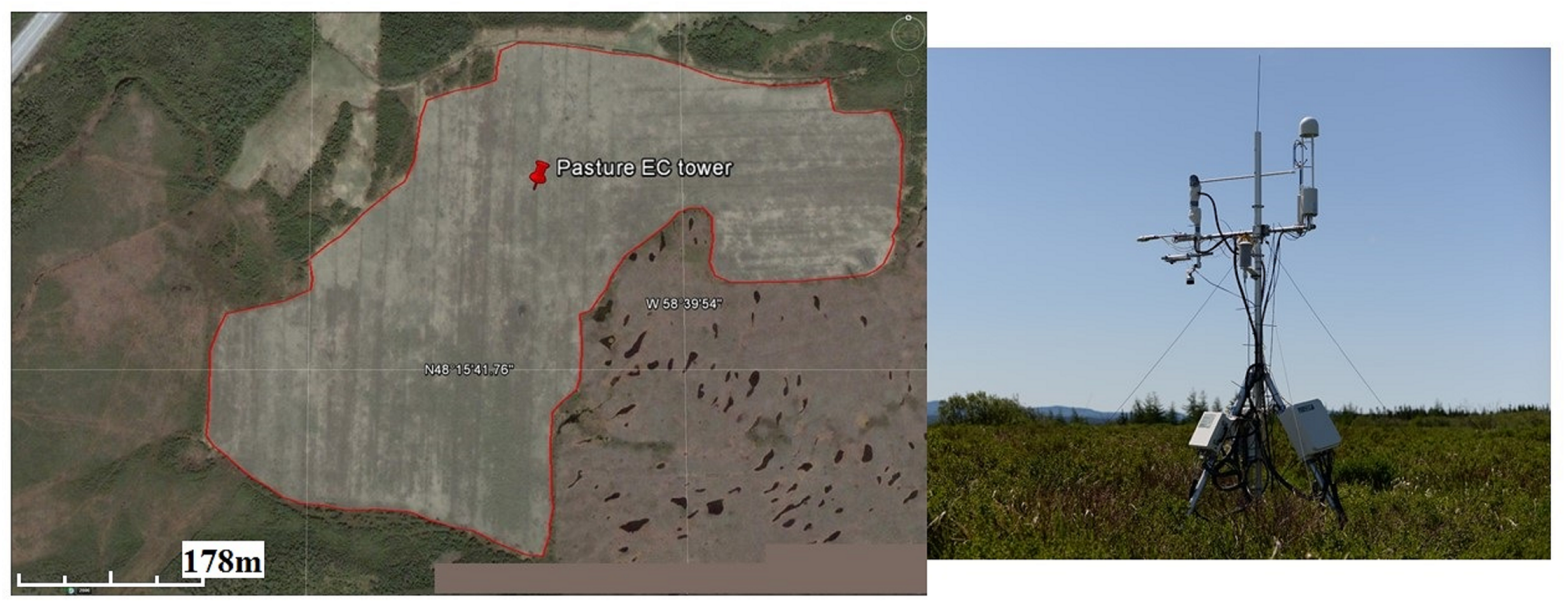
The location of flux tower in the Robinsons pasture, western Newfoundland, Canada (48.264 N, 58.665 W). The image is similar, but not identical, to the original image, and therefore is only for illustrative purposes. The outline of the site was indicated by the red solid line and the red pin represents the location of eddy covariance (EC) tower (a); (b) a photo of the setup of EC measurement system.

The study site is an abandoned peatland pasture with an average peat depth of ~4 m located in the Robinsons pasture, Newfoundland, Canada (48.264° N, 58.665° W) (No special permissions were required for these locations and our research activities, and our field studies did not involve endangered or protected species) (Fig 1). The climate is oceanic temperate with an annual temperature averaging 4.5°C and yearly rainfall of 1340 mm based on the previous 30 years’ measurements from the nearest weather station 50 km from the site. The pasture (~ 0.2 km^2^) was formerly a boreal bog that was drained by ditches in the 1970s and pasture forage grasses were introduced 35 years ago. The ditches were excavated to a depth of ~0.5 m and the width of ~30 cm along an east-west transect with a distance of 20–30 m between ditches. The site was used as pasture for 10 years and then abandoned. After the abandonment, the site was left to regenerate for ~25 years, but with continued active drainage [9,27]. In its present state, the abandoned peatland pasture is dominated by perennial grasses and shrubs, which are arranged in a mosaic of vegetation patches dominated by different species: patches dominated by reed canary grass (*Phalaris arundinacea)* and lower herbaceous and graminoid species (*Carex* spp., *Ranunculus acris*, *Ranunculus repens*, *Hieracium* sp.), and patches dominated by low shrubs, including sweet gale (*Myrica gale*), labrador tea (*Rhododendron groenlandicum*), mountain fly honeysuckle (*Lonicera villosa*), rhodora (*Rhododendron canadense*), and chokeberry (*Photinia* sp.). Despite this complex mix, there is no obvious spatial patterning in vegetation patches within the footprint of the EC tower. Plant characteristics were measured in a separate study in 2013, where peak aboveground biomass ranged from 225 to 591 g m^-2^ and root biomass varied from 186 to 340 g m^-2^ among different patches [27].

### Flux and meteorological measurements

The CH_4_ EC system consisted of a three-dimensional sonic anemometer (Gill WindMaster, Gill Instruments Ltd, Lymington, Hampshire, UK) to measure the vertical and horizontal wind vectors, and an open path infrared gas analyzer (LI-7700, LI-COR Inc., Nebraska, USA) to measure CH_4_ concentration (Fig 1). The LI-7700 and anemometer were mounted at a height of 3.6 m from the ground surface, with the northward, eastward and vertical separation from sonic anemometer of 18 cm, -1cm, and 10 cm, respectively. Data output from the EC system were recorded at 10 Hz with a data logger (LI-7550, LI-COR Inc., Nebraska, USA) and stored on a removable USB.

A set of meteorological instruments mounted on the EC system tower were used to continuously monitor environmental factors. Two quantum sensors (LI-190SL-50, LI-COR Inc., Nebraska, USA) measured the photosynthetically active photon flux density (PPFD), with the upper one measuring the incoming PPFD and the lower one the reflected PPFD. Air temperature (T_a_) and relative humidity (RH) were measured with an air temperature and humidity probe, which was installed within a ventilated radiation shield (HMP155, Vaisala, Vantaa, Finland). A tipping-bucket rain gauge mounted on the ground was used to measure total event rainfall recorded at 30-min intervals (TR-525USW, Texas Electronics, Texas, USA). Soil temperature (Ts) was measured at 1 cm, 5 cm, 10cm, 30 cm, 50 cm, and 100 cm (LI7900-180, LI-COR Inc., Nebraska, USA) and soil moisture was measured as volumetric water content at 5 cm, 10 cm, 30 cm and 50 cm below the peat surface (Delta-TML2x, Delta-T Devices, Cambridge, UK). Water table (WT) was monitored by a stainless steel transducer pressure sensor with SDI-12/RS232 connection (CS451, Campbell Scientific, Utah, USA). A four-way net radiometer was mounted at 3.6 m height to measure incoming and reflected short-wave solar radiation and incoming and emitted long-wave radiation (CNR4, Kipp & Zonen, Delft, the Netherlands). All meteorological sensors, except for the rain gauge, were scanned at 5-s intervals and recorded as half-hourly means by a data logger (CR3000-XT, Campbell Scientific, Utah, USA) located in an insulated, heated and air-conditioned instrument hut.

### Data processing

EddyPro 5.2.1 software (LI-COR, Lincoln, NE, USA) was used to process the 10 Hz raw data and output the corrected CH_4_ flux over a 30-min interval. We used the default settings for statistical tests for raw high-frequency data (despiking) [28], block averaging detrending, correction for frequency response [analytic high-pass filtering correction: [29]; low-pass filtering correction, select and configure: [29]], density fluctuations [30], sonic anemometer tilt correction with double rotation [31], angle-of-attack correction for wind components [32], lag minimization using maximum covariance with default lag of 0, and calculation of friction velocity (u*) using both along and cross wind shear. Footprint lengths were calculated following [33] and quality flags for the flux calculation were determined following [34]. For high/low pass filtering, the correction procedure is described in detail in the EddyPro manual [35], which is briefly reiterated here. Both high-pass and low-pass filtering corrections included four steps: 1) estimation of the true cospectra using a modification of the Kaimal formulation [36], 2) determining the high/low-pass transfer function (HPTF, LPTF) which is specified by the superimposition of a set of transfer functions describing sources of high/low frequency losses, 3) estimating flux attenuation by “applying” the calculated HPTF/LPTF to the modeled flux cospectra, and 4) calculating a high/low-pass spectral correction factor. For quality control and flagging, a steady state test that compares the statistical parameters determined for the averaging period and for short intervals within this period and an integral turbulence characteristics test that compares the measured parameters and the modeled ones were applied. The deviation (%) of both the steady state and integral turbulence characteristics of less than 30 indicates good data quality, between 30 and 100 moderate quality and larger than 100 bad quality. The diagnostic flag related to data quality were output, with the values of 0, 1, 2, representing data with high, intermediate, and poor quality, respectively. Further details of quality controls can be found in [37].

**Fig 2:**
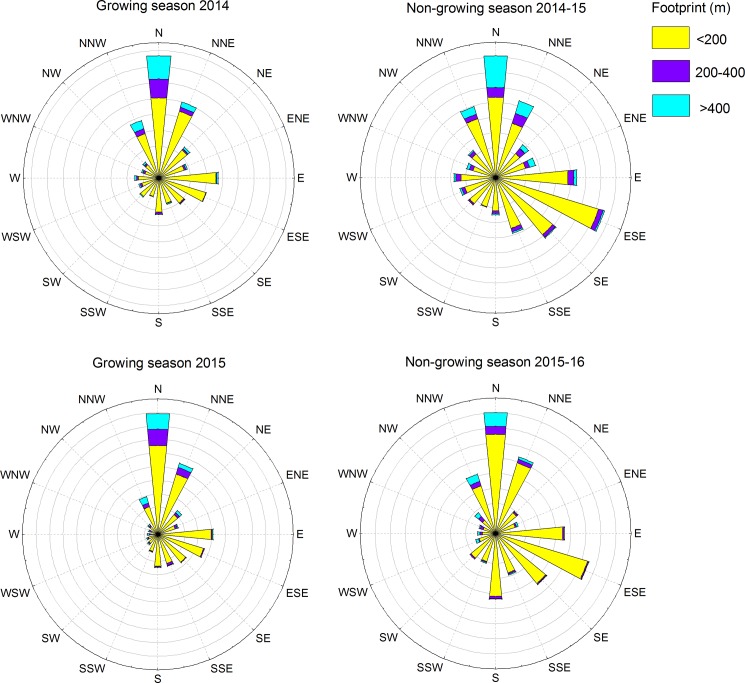
Footprint versus wind direction for different periods in the two study years. The legends indicate the cumulative footprint where 70% flux were originated. The yellow, purple and blue boxes indicate varying distances from the tower where the 70% of CH_4_ fluxes were originated.

The outputted half-hourly fluxes were corrected for spectral attenuations, air density fluctuations and instrument-specific effects as mentioned above. The magnitude of such correction factors were 1.06 and 1.12 in the growing season, 1.08 and 2.31 in the freezing period, 1.02 and 1.05 in the thawing period and 0.02 and 0.92 in the wintertime of the two study years. Flux data with a quality flag of 2 and a mean value of received signal strength indicator (RSSI) for the LI-7700 smaller than 20% were discarded. Fetch for the site varied from about 170–370 m in different directions (0–45°: 200 m; 45–77°: 287 m; 77–115°: 370 m; 115–160°: 170 m; 160–250°: 360 m; 250–360°: 200 m), so we discarded the flux data with the 70% cumulative footprint larger than these fetches. The footprints were mostly within 200 m during the different periods of both measurement years, but the dominant wind directions showed some differences among different periods (Fig 2). The dominant wind direction was from NNW to NNE during all seasons (Fig 2). We did not find a correlation between CH_4_ flux and u*, thus failing to determine a u* threshold. Therefore, we set the threshold at 0.1 m s^-1^ as in a previous study where no u* threshold could be found [38]. Flux data with u* below 0.1 m s^-1^ were discarded. The final flux data were corrected by adding the storage flux value below the height of the EC instruments. The storage flux was estimated from temporal changes in gas concentrations based on concentrations from the LI-7700 and the height integral between the instrument and peatland surface [35], under the assumption that CH_4_ concentrations were invariant with height. The CH_4_ storage flux at this site was not highly variable and was one or two orders of magnitude less than the corresponding eddy flux values.

We divided the data into growing season and non-growing season. The purpose of this division was to estimate the contribution of cumulative CH_4_ flux in each period to the annual flux budget as well as to examine the variations in the controlling factors of CH_4_ flux in each season. We further divided non-growing season into soil thawing, soil freezing and winter to investigate whether large CH_4_ bursts existed or not in the soil thawing and freezing period. We assumed growing season began and ended after the first seven consecutive days with daily air temperature above 5°C and below 5°C, respectively. We divided the growing season into three sub-periods of early growing season (May and June), peak growing season (July and August) and late growing season (September, October and November). Soil freezing ranged from the end of the growing season to the first two consecutive days with average daily soil temperature below 0°C at 10 cm depth. Winter started at the end of the soil freezing period and ended when snow started melting (after seven consecutive days with average air temperature above 0°C). The soil thawing period was between the end of the winter period and the start of the growing season.

Most of the CH_4_ flux data gaps were caused by power failures in extremely harsh weather and equipment failures, which resulted in a loss of 29% of the total flux record between May 2014 and April 2016. In addition, CH_4_ flux data were discarded due to quality control, the u* threshold and footprint filtering, thus causing additional data gaps. Overall, during the growing season data gaps of CH_4_ flux accounted for 43% and 35% of the total record in 2014 and 2015, respectively. During the non-growing periods 58% and 68% of the data were missing in 2014/15 and 2015/16, respectively.

Currently, there is no consensus on gap-filling methods for CH_4_ flux data [10,39–45]. Here we employed an artificial neural network (ANN) to fill the CH_4_ flux gaps and this method was one of a suite of tools being used for gap-filling in flux studies [46,47]. We selected the ANN method because it has been shown recently to be highly successful for gap-filling CH_4_ fluxes [39]. We used the neural network Fitting Tool in the mathematical software Matlab to select data, create and train the network, and evaluate its performance using mean square error and regression analysis. Neural networks included an input layer, a hidden layer and an output layer [48,49]and this two-layer feed-forward network with sigmoid hidden neurons and linear output neurons can fit multi-dimensional mapping problems arbitrarily well. Data were randomly divided into three sets: 70% of all data for training, 15% for testing and 15% for validating. Training data were presented to the network during training and the network was adjusted according to its error; validation data were used to measure network generalization, and to halt training when generalization stopped improving; testing data had no effect on training and so provided an independent measure of network performance during and after training. The network was trained with a Levenberg-Marquard back-propagation algorithm (trainlm) as used in previous studies [39,50]. We chose input variables including air temperature, surface soil temperature, subsurface soil temperature, PPFD, vapor pressure deficit (VPD), u* and water table (WT) according to [39]. However, during some period in wintertime, VPD and u* data were also missing, so we only used the remaining variables at these times. To set a reliable number of neurons in the hidden layer, we applied 1–10 neurons to standardized approaches [51]. The training distribution showed a constant increase in correlation coefficient with increase in the number of neurons. Therefore, we set the number of neurons in the fitting network’s hidden layer as 10. This procedure was replicated for 20 times and the median predictions were used to fill missing half-hour fluxes. Before training, all data were normalized between 0–1 [39,52–54] and divided into nighttime and daytime data sets according to a PPFD threshold of 20 μmol m^-2^ s^-1^. The gap-filled data were only used to calculate the total CH_4_ flux during each period. All analyses presented below used measured data only, except for seasonal and annual totals of CH_4_ flux, which were gap-filled. Fluxes away from the surface (i.e. CH_4_ emissions) were treated as positive and fluxes into the surface (i.e. CH_4_ sinks) were negative.

### Uncertainty estimation

Although there are many uncertainty sources in flux estimation measured by eddy covariance, here we focused on flux random uncertainty due to sampling errors, and the flux uncertainty due to the gap-filling. The other uncertainty sources can be avoided due to either carefully and properly field experiment design [55] or data processing correction, thus sampling error will remain as one of the largest sources of uncertainty. Flux random uncertainty (σ_1_) due to sampling errors is calculated following [56] in EddyPro. We estimated the flux uncertainty due to gap-filling (σ_2_) based on the following procedures. Firstly, we developed, trained and validated ANN model using the available measured data in each study period (i.e., growing season, soil freezing period, soil thawing period and wintertime). Secondly, we ran the ANN model and produced a continuous series of data for the whole two-year study period. Finally, we compared the difference between the available measured data and their counterpart predicted CH_4_ flux values from ANN model in each study period [46]. σ_2_ = 1 / N∑(*P*_*i*_−*O*_*i*_). N is the number of available measured and predicted CH_4_ flux pairs in each study period and *P*_i_ and *O*_*i*_ are the individual predicted and observed CH_4_ flux data, respectively. The total uncertainty was calculated following the equation: σ = [σ_1_^2^ + σ_2_^2^]^1/2^.

### Statistical analyses

Stepwise multivariable regression analysis was conducted to examine the effect of abiotic and biotic variables and their combined effects on CH_4_ flux, including air temperature (T_a_), surface soil temperature at 10 cm (T_10_) and subsurface soil temperature at 50 cm (T_50_), VPD, WT, PPFD, u*, soil water content at 10 cm and 50 cm (SWC_10_, SWC_50_), gross primary productivity (GPP) and net ecosystem exchange (NEE) [57]. We examined if there were significant interactions (P<0.05) among the variables before they were included in the model. The variance inflation factor (VIF) was used to test the assumption of multicollinearity. We adopted the common rule of thumb that there would be no potential multicollinearity problem if the VIF is not greater than 5 [58]. This analysis was conducted using the statistical program SAS v9.1. All data were normalized as 0–1 to approximately achieve a normal distribution before the analysis following the equation: Normalized values = (data- mindata) / (maxdata-mindata), where mindata and maxdata are the minimum and maximum value of each variable.

## Results

### Environmental conditions

The air temperature was close to the normal for most months during our study (all values within one standard deviation of the respective 30-years means), with the exceptions of warmer conditions in July 2014 and September 2015 and colder than normal conditions in March, April, June, July and November 2015 and April 2016 (Table 1). Low rainfall in September 2015 was notable, but higher than normal precipitation occurred in three consecutive winter months from November 2014 to January 2015 (Table 1).

**Table 1:** Comparison of monthly average temperature and cumulative monthly rainfall measured at Robinson Pasture during measurement periods from April, 2014 to May, 2016 with the long-term (1981–2010 average ± SD) measurements from the nearby climate station in Stephenville, Newfoundland and Labrador.

Month	Rainfall (mm)				Air temperature (°C)			
	2014	2015	2016	1981–2010	2014	2015	2016	1981–2010
Jan		54	14	29 ± 24		-6.8	-5.3	-6 ± 1.6
Feb		20	45	27 ± 30		-9.2	-3.9	-6.7 ± 2.9
Mar		12	30	37 ± 29		-6.9	-5.0	-3.5 ± 2.5
Apr		41	88	62 ± 42	1.6	-0.8	0.6	2.6 ± 1.8
May	129	118	106	94 ± 44	6.5	7.1	7.4	7.6 ± 1.4
Jun	65	64		104 ± 45	12.2	10.2		12.1 ± 1.3
Jul	97	119		118 ± 45	19.0	14.1		16.4 ± 1.1
Aug	105	125		130 ± 65	16.5	17.9		16.7 ± 0.9
Sep	83	55		128 ± 48	12.2	13.7		12.8 ± 1.1
Oct	85	101		124 ± 45	8.5	6.4		7.4 ± 1.3
Nov	133	82		94 ± 31	1.5	1.2		2.7 ± 1.3
Dec	105	54		49 ± 42	-1.5	-2.1		-2.4 ± 1.7
Overall		845		995 ± 133		3.7		5.0 ± 1

**Fig 3:**
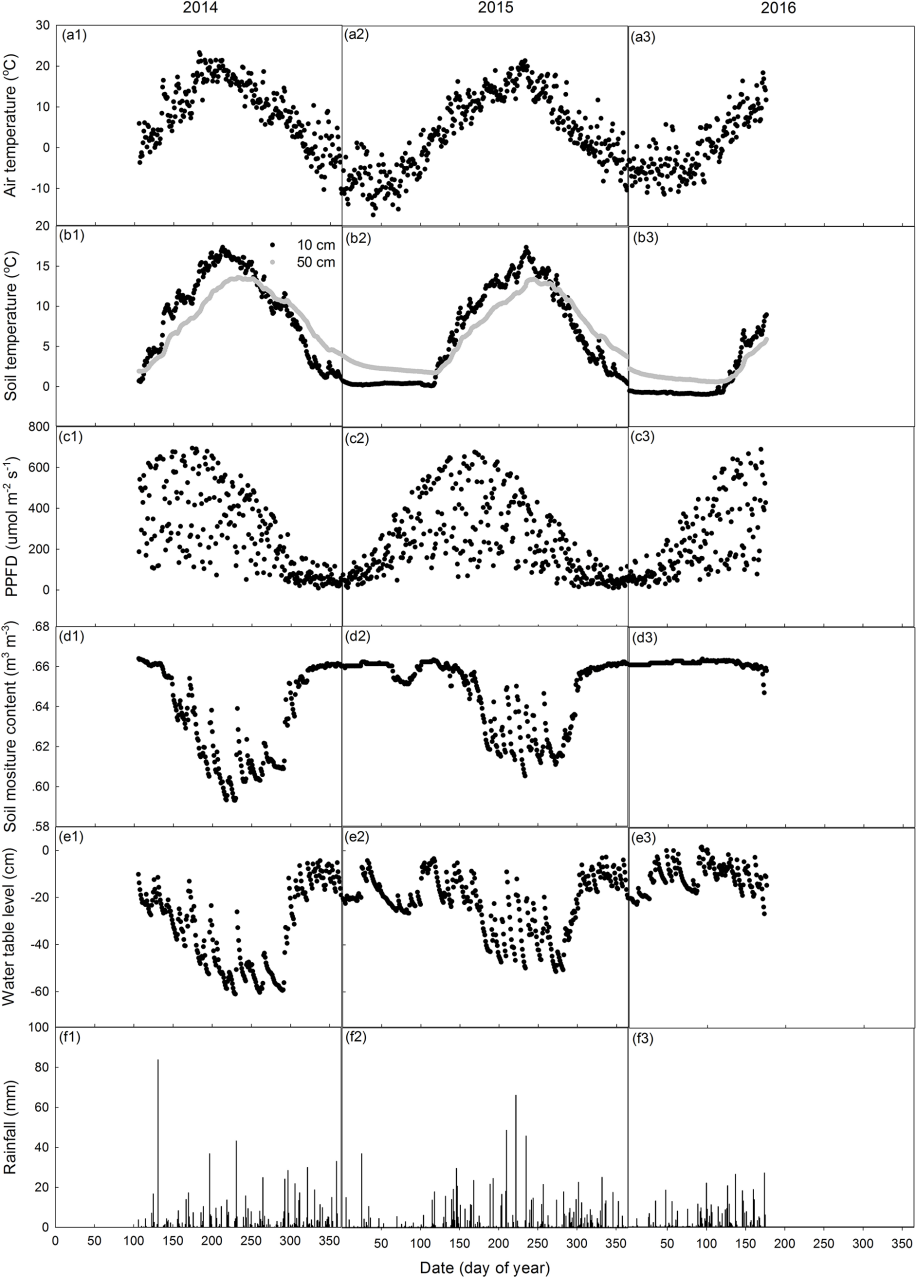
The daily average air temperature (a1-a3), soil temperature at 10 cm and 50 cm (b1-b3), photosynthetic photon flux density (PPFD) (c1-c3), volumetric soil water content at depth of 10 cm (d1-d3), water table level (e1-e3) and cumulative rainfall (f1-f3) during the measurement periods.

Environmental variables for the two study years followed typical seasonal patterns (Fig 3). The daily average air temperature ranged from ~-14.9°C to 23.2°C in the first study year and from ~ -11°C to ~21°C during the second study year, and the lowest values of both years occurred during middle-late February, while air temperature peaked in early July in 2014 and near the middle of August in 2015 (Fig 3: a1-a3). The daily average surface soil temperature at 10 cm ranged from 0.2°C to 17.3°C in 2014–15 and from -0.13 to 17.4 in 2015–16, with the lowest values occurring near the end of December when the freezing period ended and winter period began. The highest values coincided with the peak in air temperature in each year (Fig 3: b1-b3). For subsurface soil temperature at 50 cm, the seasonal trend for both years was quite similar, except with the peak delayed by 20 days in 2014 and 10 days in 2015 compared to the peak of soil surface temperature at 1 cm (Fig 3: b1-b3). The daily cumulative rainfall ranged from 0 mm to 89 mm in the first study year and from 0 mm to 53 mm in the second study year (Fig 3: f1-f3). Soil water content at 10 cm remained in a narrow range between 0.60 and 0.67 m^3^ m^-3^ in both study years (Fig 3: d1-d3). Water table was always below the peatland surface in the first year, ranging from -61 to -3 cm, with a mean value of -28.4 cm, and ranged from -52 to 2 cm in the second year with a mean of -20.5 cm, when it was slightly above the peatland surface only in April 2016 (Fig 3: e1-e3, Table 2). Although soil moisture and WT were high in the non-growing season and decreased to minimum values during the mid-growing season, both variables showed periodic sharp rises and decreases corresponding to summer rain events greater than 10 mm and the subsequent drawdowns (Fig 3: e1-e3). Mean growing season water table positions for the two years were -41.6 cm and -29.4 cm for 2014 and 2015, respectively (Table 2).

**Table 2:** Average daily air temperature, soil temperature at depth of 10 cm and 50 cm, photosynthetic photon flux density (PPFD), cumulative rainfall, and water table position for four different periods. Negative values indicate water table was below the peatland surface.

Period	Date	Air temperature	Soil temperature (°C)		PPFD	Rainfall	Water table
		(°C)	10 cm	50 cm	(mol m^-2^ d^-1^)	(mm)	(cm)
Growing season	2014.5.15–11.11	12.8	12.3	10.5	28.7	513	-41.6
	2015.5.16–11.15	11.4	11.1	9.9	25.4	603	-29.4
Soil freezing	2014.11.12–12.28	-0.4	2.5	5.8	4.9	176	-11.5
	2015.11.16–12.29	-1	2.4	5.4	5	92	-10.9
Winter	2014.12.29–2015.5.3	-5.8	0.5	2.4	18.2	126	-17.1
	2015.12.30–2016.5.1	-3.4	0.1	2.3	15.1	174	-11.1
Soil thawing	2014.5.1–5.14	2.6	4.3	3.3	35.1	112	-19.6
	2015.5.2–5.15	5.7	3.1	2.4	29.2	38	-7.7
Overall	2014.5–2015.5	4.3	6.8	6.7	22.7	936	-28.4
	2015.5–2016.5	4.6	6.0	6.5	20	890	-20.5

### Seasonal dynamics of CH_4_ fluxes

**Fig 4:**
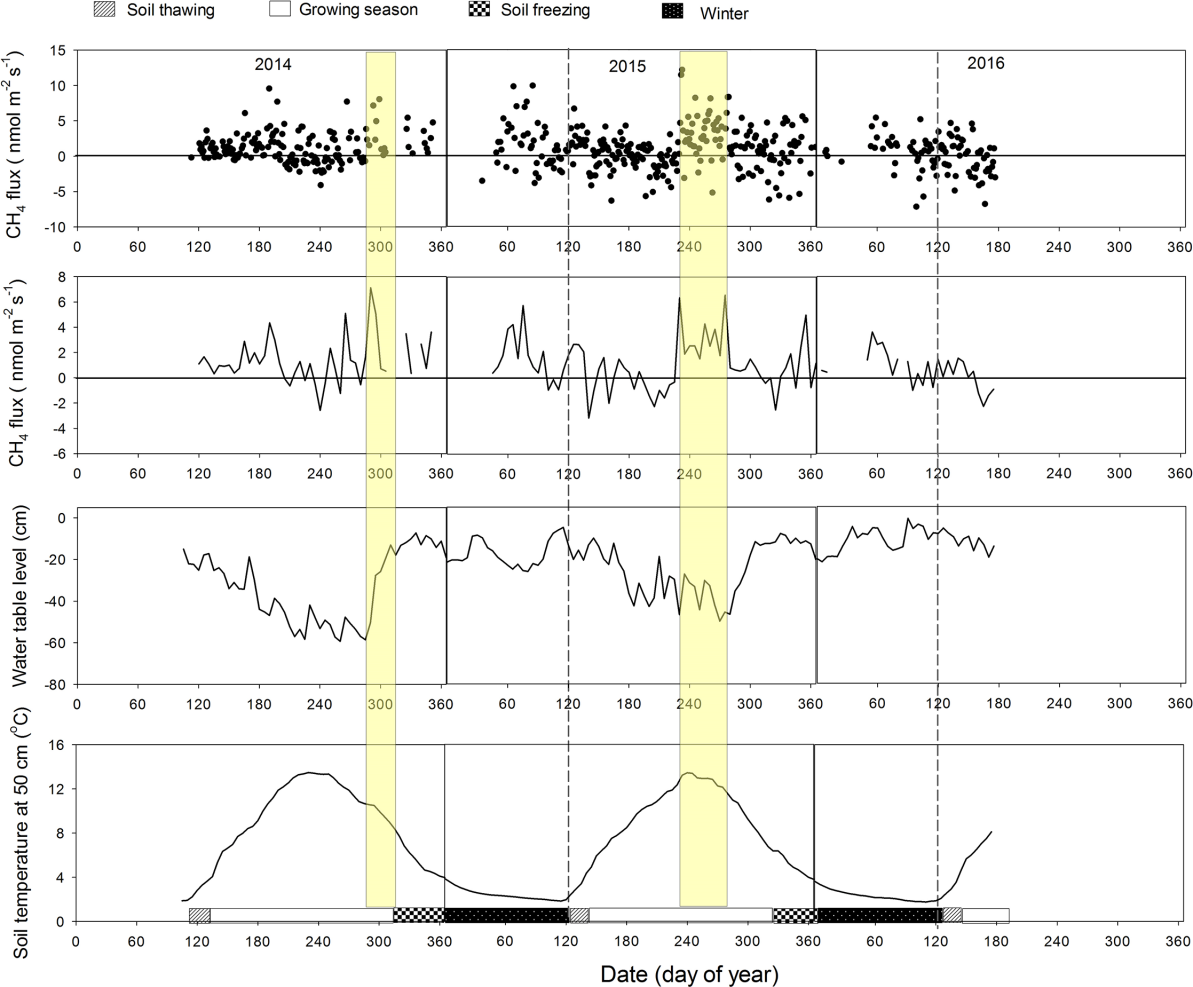
The daily average CH_4_ flux (a), five-day running average CH_4_ flux (b), five-day running average water table level (c) and five-day running average soil temperature at 50 cm (d) of different periods during the two study years.

There was no clear seasonal pattern in CH_4_ fluxes in either study year, even when smoothed with a 5-d running average (Fig 4). Some pronounced periods of emissions occurred in the late growing season at DOY280-310 in 2014 corresponding to the increase in WT and DOY 230–270 in 2015 coincident with high T_50_ (Fig 4). Further, we did not find a large CH_4_ burst during soil freezing and thawing periods and CH_4_ uptake was observed in all seasons in both years. In general, the CH_4_ fluxes were small, varying around zero with the daily average CH_4_ flux ranging from -4.1 to 9.9 nmol m^-2^ s^-1^ over the first study year and from -7.1 to 12.1 nmol m^-2^ s^-1^ over the second study year (Fig 4). The range of wintertime CH_4_ emission fluxes was comparable to that of the growing season. On a seasonal basis cumulative CH_4_ showed emissions in most seasons, except the soil freezing period when cumulative uptake was recorded in both years (Table 3).

**Table 3:** Total accumulated CH_4_ fluxes, their uncertainties (g CH_4_ m^-2^) for the different study periods and contributions to the annual emissions in two years from May 2014 to April 2016. RU, GU and TU in the table indicate random uncertainty, uncertainty due to gap filling and total uncertainty, respectively.

	From May 2014 to April 2015							From May 2015 to April 2016						
Period	Duration days	CH_4_ flux	RU	Ratio of RU	GU	Ratio of GU	TU	Duration days	CH_4_ flux	RU	Ratio of RU	GU	Ratio of GU	TU
				to flux		to flux					to flux		to flux	
Growing season	181	0.17	0.25	1.49	0.003	0.02	0.25	184	0.27	0.32	1.15	0.01	0.03	0.32
Soil freezing	47	-0.04	0.02	0.59	0.002	0.05	0.02	44	-0.27	0.19	0.7	0.05	0.02	0.19
Winter	125	0.25	0.16	0.65	0.03	0.12	0.16	124	0.1	0.08	0.75	0.03	0.3	0.08
Soil thawing	12	-0.02	0.04	2.11	0.01	0.53	0.04	13	0.03	0.04	1.35	0.002	0.08	0.04
Annual Total	365	0.36	0.3	2.73	0.03	0.09	0.3	365	0.13	0.38	2.05	0.03	0.24	0.38

**Fig 5:**
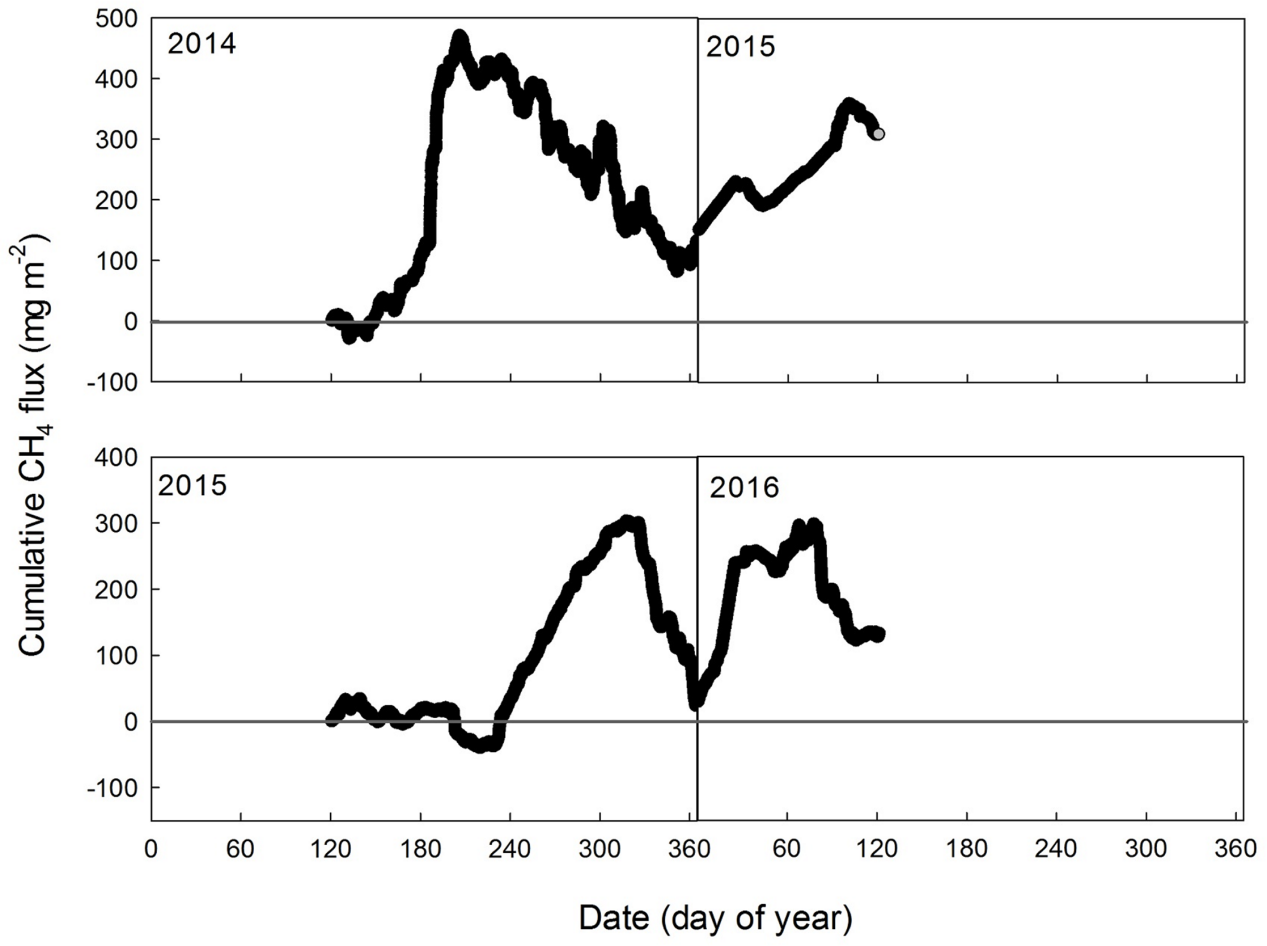
The cumulative gap-filled CH_4_ flux during the two study years (from May 2014 to April 2016).

Although our annual CH_4_ flux estimates suggested this abandoned pasture was a net source of CH_4_ to the atmosphere, the annual totals were not significantly different from zero at 0.36 ± 0.30 g CH_4_ m^-2^ yr^-1^ in 2014–15 and 0.13 ± 0.38 g CH_4_ m^-2^ yr^-1^ in 2015–16 (Fig 5, Table 3). The largest uncertainty in the annual estimates came from random errors of 0.30 g CH_4_ m^-2^ yr^-1^ in 2014–15 and 0.38 g CH_4_ m^-2^ yr^-1^ in 2015–16 (Table 3). The flux bias associated with the gap-filling was neglected during the growing season in both years since the agreement between modeled and measured CH_4_ fluxes was high (i.e., model efficiency >80%). However, a low model efficiency of 20% was found during the non-growing season due to the lack of strong dependence of CH_4_ flux on environmental variables. As a result, the uncertainty due to the gap-filling was pronounced during wintertime of both study years and soil thawing period in 2014–15, with the bias accounting for 12% -53% of the accumulated flux (Table 3).

Results from the multiple regression analysis showed shifting controls on CH_4_ flux over different growing season periods and years (Table 4). During the early growing season, CH_4_ flux was closely related to CO_2_ fixation rate in both study years; VPD and T_a_ exerted a positive effect on CH_4_ flux in 2014 and 2015, respectively. During the peak growing season, CH_4_ flux increased and decreased with the increase in SWC_10_ and T_50_, respectively, in 2014. In 2015, CH_4_ flux increased with the increase in T_50_ and u*. During the late growing season, WT exerted a positive effect on CH_4_ flux, while T_a_ affected CH_4_ flux negatively in 2014 and no significant correlation was found in 2015. For the whole growing season, although WT and soil temperature exerted some impact on CH_4_ flux, only less than 10% of the variation in CH_4_ flux can be explained by WT and soil temperature in both study years, which together with PPFD explained only 10% of the variation in CH_4_ flux in 2014, and together with VPD and SWC_10_ explained 13% of the variation in CH_4_ flux in 2015 (Table 4).

**Table 4:** The results of stepwise multivariable regression analysis between daily average CH_4_ flux and abiotic variables including friction velocity (u*), vapor pressure deficit (VPD), photosynthetically active photon flux density (PPFD), air temperature (T_a_), soil temperature at 10 cm and 50 cm (T_10_, T_50_), soil water content at 10 cm and 50 cm (SWC_10_, SWC_50_) and water table level (WT) and biotic variables such as gross primary productivity (GPP) and net ecosystem exchange (NEE). Only significant (P<0.05) variables were included in the equation. No significant interactions among the variables were found (P>0.05), and the variance inflation factor (VIF) for all variables in the model is less than 5.

Period	Sub-period	Year	Model	Total R^2^	*P*	df
Growing season	Early	2014	Y = -0.05 + 0.0005 VPD + 0.0004GPP	0.4	0.001	34
		2015	Y = -0.006–0.0007NEE + 0.002 T_a_	0.2	0.009	45
	Peak	2014	Y = 0.036–0.008T_50_ + 0.032SWC_10_	0.25	0.0003	58
		2015	Y = 0.27 + 0.001u*+ 0.40T_50_	0.25	0.0003	58
	Late	2014	Y = 0.005 + 0.002 WT– 0.003T_a_	0.31	0.0004	60
		2015				
	All Periods combined	2014	Y = -0.006 + 0.001WT + 0.003T_50_-0.0005PPFD	0.10	0.0025	154
		2015	Y = -0.02 + 0.004T_50_ + 0.001VPD—0.003 T10−0.02 SWC_10_	0.13	<0.0001	177

## Discussion

### Controls on the seasonal dynamics of CH_4_ flux

Although our analysis suggested that WT and soil temperature exerted some impact on growing season CH_4_ flux in both study years, the correlations were not strong (R^2^: 0.1~ 0.25, Table 4). This result is similar to the findings at drained peatlands elsewhere [10,59,60]. The low CH_4_ emission rates contribute to the lack of a strong seasonal pattern in CH_4_ flux, as well as the lack of consistency in the underlying controls [59,60]. Moreover, we found some pronounced emissions in the late-growing season in both study years (Fig 4), which was shown to be correlated with the rapid increase in WT in 2014 but coincident with the high T_50_ in 2015. The difference was probably due to the different hydrologic conditions of the late growing season in the two years, with more rapid increase in WT in 2015 than that in 2014 (Fig 3). It has been suggested elsewhere that WT was a principal control on CH_4_ flux when the WT was very low, while soil temperature became dominant when the WT was higher [61].

Our data indicated that the controls on CH_4_ flux varied among different seasonal periods. During the early growing season, CH_4_ flux was found to be closely related to the variation in NEE and GPP, suggesting that substrate availability was a limiting factor in determining CH_4_ flux. The newly absorbed C via photosynthesis (i.e. GPP) can be transferred to either root exudates or fresh litters and thus affects the quantity and quality of substrate for methanogenic activities. Luan and Wu (2015) [9] found that the variation in substrate availability explained 15–20% of the variation in CH_4_ emissions at the same site by using dissolved organic carbon (DOC) as a proxy of substrate availability. They found that the variation in DOC was primarily regulated by the changes in GPP. Although substrate availability has been recognized as an important control on CH_4_ fluxes in other northern peatlands [16,62–67], our finding highlights the importance of substrate availability in regulating CH_4_ flux during substrate-limited periods. During the early growing season, plants have not fully developed yet to produce enough fresh litter and root exudates for CH_4_ production, and thus the primary constraint for CH_4_ flux is due to the limitation of high quality C resources. In addition, early growing season CH_4_ flux was also related to the variation in VPD, implying that CH_4_ flux was also regulated by the flux transport process as suggested by Tripathee (2014) [68]. The increase in VPD results in opening of the stomata and increased transpiration [69], which will promote the plant-mediated CH_4_ transport to the atmosphere and thus increase CH_4_ emissions [70].

During the peak growing season, CH_4_ flux responded differently to the variations in T_50_ in the two study years, which we assume was mainly due to the different WT conditions. The peak growing season WT averaged -48 cm (-24 ~ -61 cm) in 2014, lower than that of -35 cm (-9 ~ -50 cm) in 2015, and the low WT in 2014 may have enhanced the role of CH_4_ oxidation in regulating CH_4_ flux. Indeed, we found that CH_4_ flux was negatively related to T_50_ but positively to SWC_10_ in 2014 peak growing season, suggesting that CH_4_ flux was mainly determined by the oxidation process. Both process-level and field studies have identified soil temperature and soil moisture as key controls on CH_4_-oxidation in soils [71–74], with increasing soil temperature promoting the CH_4_ oxidation via stimulating methanotrophy activity, but increasing SWC inhibiting CH_4_ oxidation by decreasing the oxygen availability in soils. In 2015, more frequent rises of the WT following large rainfall events were observed [Fig 3 (e2)], enhancing the role of CH_4_ production in determining the CH_4_ flux, resulting in a positive CH_4_-temperature relationship (Table 4). Overall, our result suggested that neither soil temperature nor WT/SWC come out as a dominant factor in most models and sometimes they have different signs in different years, implying the interacting effects of CH_4_ production and consumption can cancel each other out.

During the late growing season, we found that CH_4_ flux was positively related to WT in 2014, which was similar to many previous findings that CH_4_ emission rate increased with the increase in WT [20,75,76]. The positive effect of WT on CH_4_ flux can also serve to further explain the pronounced emissions in the late growing season (Fig 4). Two mechanisms may exist to explain the CH_4_ emission spike as a result of a sudden rise of water table. Firstly, the abrupt CH_4_ emission could be due to the previously stored CH_4_ in the soil matrix that is abruptly emitted to the atmosphere as water table rises. Secondly, the enhanced CH_4_ production because of a sudden rise of water table could also suddenly increase CH_4_ emissions to the atmosphere. However, we did not have direct evidence to tease out which mechanism would be the dominant mechanism. Therefore, more mechanism-based process studies are needed to examine the mechanism behind this phenomenon and the relative contribution from either mechanism.

### Comparison of long-term CH_4_ flux with other peatland pastures

With a few exceptions CH_4_ flux from managed peatlands has been considered to be insignificant for the annual greenhouse gas balance [4,22,24,60,77–79]. We found that the total annual CH_4_ emissions were small and not significantly different from zero in the two study years (0.36 ± 0.30 g CH_4_ m^-2^ yr^-1^ in 2014–15 and 0.13 ± 0.38 g CH_4_ m^-2^ yr^-1^ in 2015–16). These values are similar to the range of annual fluxes from managed peatlands in European countries and Canada (-0.17–1.6 g CH_4_ m^-2^ yr^-1^), but lower than the 11.4 g CH_4_ m^-2^ yr^-1^ observed in California, USA and the 14.6–20.3 g CH_4_ m^-2^ yr^-1^ measured in the Netherlands (Table 5). In these latter two cases, the high CH_4_ mission rates were attributed to relatively high temperatures throughout the year at the California site [80] and the continuous application of decomposable organic materials which improved the substrate for methane production at the Dutch pasture [19,81]. Moreover, the low growing season CH_4_ emission rates of ~0.2–0.3 g CH_4_ m^-2^ we observed in 2014 and 2015 were similar to a growing season rate of ~1 g CH_4_ m^-2^ based on chamber measurements at our site in 2013 [9]. These fluxes are within the range of -0.18 to 1.1 g CH_4_ m^-2^ per growing season measured elsewhere in managed peatlands (Table 5). In addition, we found that the CH_4_ emission rates (mostly less than 1 g CH_4_ m^-2^ yr^-1^) from agriculturally managed peatlands were much lower than that of ~27 g CH_4_ m^-2^ yr^-1^ for natural peatlands (Table 5). We attribute the low emissions at the agriculturally managed peatlands to the relatively thick aerobic layer resulting from the low WT, which averaged ~ -70 cm (-30 ~ -110 cm), much lower (~-43 to -10 cm) than that of natural peatlands. We assume that CH_4_ produced in the anaerobic layer below the WT was largely oxidized before being emitted to the atmosphere, resulting in extremely low emissions for the agriculturally managed peatlands [4,9,22,24,60,77–79]. CH_4_ uptake was observed in all seasons at this site, which is not unusual in managed peatland systems. For example, growing season CH_4_ uptake was found at an intensively managed grass peatland in the Netherlands [82] and at a fen drained and converted to grassland in Finland [59].

**Table 5:** Comparison of accumulated methane flux balance for agriculturally managed peatlands and natural peatlands.

	Location				Peatland type	Study Method	CH_4_ flux (g CH_4_ m^-2^ yr^-1^)		WT	Ref.
	Country	Province/City	Latitude°N	Longitude°E			Growing season	Annual average	cm	
Agriculturally managed peatlands	Finland	Markku Lappalainen	62.67	30.83	Drained for grass	Chamber	-0.17	0.13	-70	[59]
	Finland	Jokioinen	60.82	23.5	Drained for grass	Chamber	-0.18~-0.08	-0.17~0.64	-110	
										[4,60,78,79,83,84]
	Sweden	Västra Götaland	58.33	13.5	Drained for grass	Chamber	0.09	0.12	-58	[22]
	Norway	Bodø	67.28	14.47	Drained for grass	Chamber		1.5~1.6		[24,77]
	Netherland	South Holland	52.03	4.77	Drained for grass	Eddy covariance & Chamber		14.6–20.3	-50	[10,21]
	USA	California	38.1	-121.64	Drained for grass	Eddy covariance		11.4	-65	[80]
	Canada	Napierville	45.13	-73.43	Drained for crop	Chamber	-0.06~-0.08	0.2	-100	[85]
	Canada	Robinson pasture	48.26	-58.67	Drained for grass	Chamber	1.1			[9]
	Canada	Robinson pasture	48.26	-58.67	Drained for grass	Eddy covariance	0.1–0.1	0.3–0.4	-30	This study
Natural peatlands	Estonia	Pärnu	58.47	25.21	Temperate bogs	Static chamber		11.3	-9.3	[18]
	Finland	Ruovesi	61.83	24.2	Boreal fen	Eddy covariance	41.3	16.8	-10	[43]
	Finland	Lapland	69.13	27.27	Arctic mire	Eddy covariance		7.3	14	[86]
	Finland	Ilomants	62.75	-31.05	Boreal fen	Chamber		34.7	-17.5	[59]
	Germany	Swabia	47.81	-11.46	Temperate bog-pine	Eddy covariance		7.1	-5	[87]
	Poland	Łomża	53.59	22.89	Temperate mire	Eddy covariance			-29	[88]
	Russia	Komi Republic	61.93	50.22	Boreal peatland mixture	Static chamber		34.1		[89]
	Siberia	Plotnikovo	57	82	Boreal bog	Static chamber				[90]
	Sweden	Västerbotten	64.18	19.55	Boreal fen	Static chamber		12, 19	-17	[91]
	Sweden	Abisko	68.33	19.05	Subarctic palsa mire	Eddy covariance		36		[92]
	USA	Minnesota	47.51	-93.49	Temperate poor fen	Eddy covariance		21.7	0	[93]
	USA	Minnesota	47.53	-93.46	Temperate bog	Static chamber		57.3		[94]
	USA	Minnesota	47.53	-93.46	Temperate poor fen	Static chamber		87.6		[94]
	USA	Minnesota	47.32	-93.47	Temperate bog	Chamber		49.3		[95]
	USA	New Hampshire	43.21	-71.06	Temperate poor fen	Static chamber	152		-20	[96]
	USA	Michigan	46.32	-86.05	Sub-boreal	Eddy covariance	17.3		-18	[16]
	Canada	Quebec	53.68	-78.17	Boreal bog	Eddy covariance	28		-11	[97]
	Canada	Ontario	45.68	-75.8	Temperate bog	Chamber&Eddy covariance		9.3		[98]
	Canada	Ontario	45.68	-75.8	Temperate bog	Autochamber		9.5, 11.6	-13.4	[99]
	Canada	Ontario	45.68	-75.8	Temperate bog	Static chamber		4.9		[98]
	Canada	Ontario	45.68	-75.8	Temperate bog	Eddy covariance	19.5		-43	[61]
	Canada	Quebec	54.8	-66.82	Boreal fen	Static chamber	0.1		10	[100]
	Canada	Quebec	54.8	-66.82	Boreal fen	Static chamber	13.1		0	[100]
	Canada	Quebec	54.8	-66.82	Boreal rich fen	Static chamber	4		-10	[100]
	Canada	Alberta	54.82	-112.47	Boreal fen	Eddy covariance	12.4		-33	[41]

Our study was conducted at the abandoned peatland pasture with active drainage and the data indicated that annual CH_4_ emission was not significantly different from zero. This is near the lower end of the range of CH_4_ emissions observed in other agriculturally managed peatlands (Table 5). It is notable, however, that the water table at our site was relatively shallow compared to other managed peatlands (Table 5). On the other hand, water table is similar to that in many studies on undisturbed peatlands, yet our abandoned peatland pasture had a significantly lower annual CH_4_ emission (Table 5). Therefore, in terms of CH_4_ emissions, the abandonment has made this ecosystem significantly different from both actively managed peatlands and natural peatlands. More study is needed in other abandoned pastures to confirm the universality of our findings.

## Conclusion

This study updates our knowledge of the short-term variations of CH_4_ flux and its abiotic and biotic controls at an abandoned boreal peatland pasture based on high temporal-resolution CH_4_ flux data. We found the CH_4_ flux of the abandoned peatland pasture was very low, to the point they are likely not significant in the peatland’s overall C balance. This finding is consistent with previous research in agriculturally managed peatlands. The very low and errantic fluxes confounds the search for distinct temporal (diel or seasonal) patterns in the CH_4_ flux and the identification of significant environmental drivers. Our results also suggested the controls on CH_4_ flux shifted among different growing season periods, therefore different relationships should be used to model the CH_4_ flux in these environments over time.
